# Stress and Non-Stress Roles of Inflammatory Signals during HSC Emergence and Maintenance

**DOI:** 10.3389/fimmu.2016.00487

**Published:** 2016-11-07

**Authors:** Thomas Clapes, Stylianos Lefkopoulos, Eirini Trompouki

**Affiliations:** ^1^Department of Cellular and Molecular Immunology, Max Planck Institute of Immunobiology and Epigenetics, Freiburg, Germany

**Keywords:** inflammatory signaling, hematopoiesis, zebrafish, mouse, development, stress, disease

## Abstract

Hematopoietic stem cells (HSCs) are a rare population that gives rise to almost all cells of the hematopoietic system, including immune cells. Until recently, it was thought that immune cells sense inflammatory signaling and HSCs respond only secondarily to these signals. However, it was later shown that adult HSCs could directly sense and respond to inflammatory signals, resulting in a higher output of immune cells. Recent studies demonstrated that inflammatory signaling is also vital for HSC ontogeny. These signals are thought to arise in the absence of pathogens, are active during development, and indispensable for HSC formation. In contrast, during times of stress and disease, inflammatory responses can be activated and can have devastating effects on HSCs. In this review, we summarize the current knowledge about inflammatory signaling in HSC development and maintenance, as well as the endogenous molecular cues that can trigger inflammatory pathway activation. Finally, we comment of the role of inflammatory signaling in hematopoietic diseases.

## Introduction

The processes of blood formation during development and its maintenance in adulthood have been intense topics of study for many years. Special attention is given to hematopoietic stem cells (HSCs) that can replenish the hematopoietic system and play pivotal roles during development and adult homeostasis. Given their multipotential and self-renewing capacities, HSCs are the key cell type in bone marrow (BM) transplantations to treat hematological diseases. Since some malignancies are the outcome of deregulation of the hematopoietic system, it is critical to understand how HSC homeostasis is maintained under steady state, but also under various stress situations. In this review, we will discuss the role of inflammatory cytokines in hematopoietic stem cell emergence but also during adulthood summarizing the current status. We will give a brief outline of the role of these signals in disease. The detailed ontogeny and molecular control of HSCs is beyond the scope of this review, but has been described in others (1–3).

Fairly and recently, inflammatory signaling and its role in HSC formation and maintenance has been of great interest for many researchers. For many years, it was believed that HSCs could not respond directly to these signals. Many groups have now shown that different cytokines and tonic inflammatory signaling are sensed by HSCs. These signals are required for the formation of HSCs during both mouse and zebrafish development. Similar cytokines are pivotal for maintaining HSC quiescence in adult organisms and regulating their differentiation in case of stress or disease.

Hematopoiesis is conserved between model organisms and two of them have been used extensively for studying both embryonic and adult hematopoiesis: zebrafish and mouse. During embryonic development, they both experience different hematopoietic waves (4, 5). The primitive wave is only transient and gives rise mainly to erythrocytes and myeloid cells, whereas the definitive wave gives rise to the actual HSCs through the conserved endothelial-to-hematopoietic transition (EHT) (6–9). In both organisms, EHT takes place in the aorta-gonad-mesonephros (AGM) region. Whether EHT in mice also takes place in other hematopoietic organs, such as in the placenta (10) or in the head (11–13), is still under question. Soon after generation, HSCs migrate to the caudal hematopoietic tissue (CHT) in zebrafish and to the fetal liver in mouse, where they amplify. Finally, HSCs reach the adult hematopoietic organs, namely the zebrafish kidney marrow and the mouse BM, where they remain quiescent and few of them actively replenish the hematopoietic system. However, in case of stress emergency, hematopoiesis arises and HSCs take the lead in reestablishing the balance of the hematopoietic system. All studies concerning hematopoiesis in those two organisms not only confirm some basic anatomical and functional equivalences between them but also a shared genetic program.

## Interferons

Interferons (IFNs) are antiviral cytokines produced by host cells in response to pathogens (viruses, bacteria, and parasites) and tumor cells. Type I IFNs (IFN-α, IFN-β) are produced by a variety of cell types and signal through the IFN α/β receptor (IFNAR) on target cells. Type II IFN (IFN-γ) is released mainly by T-cells and NK-cells and signal *via* IFN-γ receptor (IFNγR) present on immune cells. Finally, type III IFNs include three IFN-λ (lambda) molecules, called IFN-λ1, IFN-λ2, and IFN-λ3 that signal through IL10R2 and IFNLR1 (14).

## The Impact of Interferons on Embryonic Hematopoiesis

The zebrafish mutant *crfb17*^−/−^ (ortholog of IFNGR1) demonstrated a marked reduction in HSCs budding from the ventral wall of the dorsal aorta, compared to their *crfb17*^+/+^ siblings. Consistently, morpholino-mediated knockdown of the same receptor, as well as *ifng1-2* (15) reduced the expression of an important marker of HSPCs, *runx1*, at 33 hpf (hours post fertilization), indicating that IFN-γ signaling positively regulates HSCs. Notch signaling, a known regulator of EHT (16), was not sufficient to induce the EHT process in an *ifng1-2*-deficient background proving that *ifng* acts downstream of Notch (15). Interestingly, Sawamiphak et al. also demonstrated that IFN-γ expression in the AGM is dependent on blood flow (15), another important positive regulator of HSC emergence (17). Indeed, ifng1-2 overexpression partially rescued this defect (15), hinting to an interplay between IFN signaling and mechanical signals that regulate blood flow.

This correlation between IFN-γ signaling and HSC emergence seems to be conserved in mice (18). The numbers of HSCs in mouse embryos deficient in IFN-γ or IFN-γ receptor are decreased compared to wild type embryos. At the same time, transplantable HSCs are also decreased in embryos deficient for this receptor, all together showing an important role of this cytokine in HSC emergence. The conserved IFN effect on mammalian hematopoiesis is also supported by the fact that inflammatory signaling was found active in human fetal hematopoietic stem and progenitor cells (HSPCs) (18).

Apart from type II, type I IFNs can also regulate the process of embryonic hematopoiesis in both organisms, in a manner consistent with that of IFN-γ signaling. In particular, IFN-α in mice and its ortholog, IFN-ϕ, in zebrafish positively regulate the number of HSPCs during embryonic development (18), as embryos lacking IFN-α or -γ signaling harbor fewer HSPCs in the AGM. In another mouse study, it was shown that AGM HSCs exhibit lower levels of IFN-α than fetal liver HSCs, and this attribute can account for the lower engraftment potential of AGM HSCs. Treatment with IFN-α greatly enhances the repopulation capacity of AGM-HSCs. Mechanistically, adenine-thymine-rich interactive domain-3a (Arid3a) is responsible for the regulation of Stat1 and various IFN-responsive genes in developmental hematopoiesis (19).

Last, micro RNAs have been shown to regulate IFN-responsive genes and affect HSC development. mir-142-3p is essential for HSC emergence in both zebrafish and xenopus (20, 21). As shown in zebrafish, the main function of mir-142-3p is the downregulation of *irf7* (20).

## The Impact of Interferons on Adult Hematopoiesis

For decades, the role of IFNs, and in particular IFN-γ, has been quite controversial. *In vitro* studies on human cells reported that IFN-γ had generally a suppressive effect on hematopoietic progenitors (22–25), but could synergize with other growth factors, such as IL-3, IL-6, EPO, and IL-1b to stimulate HSPC proliferation (26–28). Colony-forming assays of human progenitor cells showed that all IFNs suppressed the formation of colony-forming unit-granulocyte, erythroid, macrophage, megakaryocyte (CFU-GEMM), and burst-forming unit-erythroid (BFU-E), but granulocyte-macrophage (CFU-GM) were mostly responsive to IFNγ and less to IFNα (29, 30). Interestingly, this regulation was also influenced by oxygen levels (31).

One of the first *in vivo* evidence demonstrating that IFNs could affect the adult hematopoietic progenitor compartment arose in a report studying lymphocytic choriomeningitis virus (LCMV) infection in mice. The authors observed that LCMV infection causes depletion of hematopoietic progenitors [colony-forming unit in the spleen (CFU-S)]. Interestingly, they demonstrated that BM aplasia following LCMV infection was abrogated in mice lacking both IFN-α/β receptors (32). Other reports have also intended to delineate the role of IFNs *in vivo* using mouse model. Sato et al. demonstrated that IFN-α treatment could induce the proliferation of HSCs (side population/LSK). Moreover, the authors showed that the interferon regulatory factor 2 (IRF2), a suppressor of type I IFN receptors, was responsible for maintaining HSC quiescence (33). Consistent with these findings, Essers et al. have shown that IFN-α could facilitate the exit of HSCs (LSK CD150^+^CD48^−^) from quiescence through activation of STAT-1 and AKT (34), and possibly by the upregulation of c-Myc protein (35). Furthermore, it was shown that the tonicity of the stimulation had a great impact on HSC repopulating capacity. Whereas acute and short-term (three doses) IFN-α treatment did not impair HSC activity, chronic exposure (eight doses for 2 weeks) to IFN-α greatly compromised HSC repopulation capacity in competitive transplantation assays (34). In depth studies of the acute and chronic exposure of HSCs to type I IFNs showed that, although brief exposure leads HSCs to exit quiescence, chronic exposure leads them back to quiescence. However, upon stress, when they are forced to enter cell cycle again (i.e., by transplantation), they activate a p53-dependent proapoptotic gene program (36), which would explain the suppressive effect of type-I IFNs on HSC activity.

Besides IFN-α, IFN-γ can also enhance the proliferation of HSPCs (LSK) *in vitro* and *in vivo via* STAT1 (37). Interestingly, murine BM cells cocultured with stromal cells overexpressing IFN-γ failed to reconstitute congenic recipient mice upon transplantation (38). Mice infected with *Mycobaterium avium* have a greater proportion of proliferating HSCs (LSK CD150^+^) due to increased IFN-γ signaling. Indeed, stimulation with IFN-γ alone could also mediate HSC proliferation *in vivo*. Moreover, IFN-γ-treated HSCs had decreased repopulating capacity compared to untreated HSCs upon *in vivo* transplantation assays (39). Similarly, mice infected with *Ehrlichia muris* also harbored increased HPSC (LSK) proliferation and reduction of long-term repopulating capacity (40). Interestingly, de Bruin et al. observed that IFN-γ impaired HSC maintenance by perturbing TPO signaling *via* SOCS-1. However, the authors did not find evidence for enhanced proliferation of HSCs, but rather that IFN-γ could modulate the expression of genes involved in HSC self-renewal, such as Cyclin D1 (41). Type I IFN can also regulate emergency megakaryopoiesis by upregulating megakaryocytic proteins in HSCs that already transcribe mRNAs of the megakaryocytic program (42).

Finally, ablation of the interferon-inducible GTPase Lrg-47 (*Irgm1*) inhibits baseline HSC proliferation and leads to severe impairment of HSCs in repopulation assays (43). Molecularly, it was proven that Irgm1 is a potent negative regulator of IFN response and that is how it exerts its action on HSCs (44).

Altogether, multiple recent reports in the literature prove that IFNs regulate HSC activity. However, it is still difficult to actually compare all these studies, as markers used to characterize HSCs differ (see Table 1). It is also important to note that some markers, such as Sca-1 (*Ly6A*), are upregulated following IFN-α stimulation (34). It is therefore critical to link HSC phenotype to their actual activity, by means of *in vivo* transplantation assays. Finally, it seems that both type I and II IFNs appear to have similar effects on HSCs. However, it is still unclear whether both types of IFN have redundant function in activating HSCs upon infection. It is likely that they are activated by specific cues, but concrete evidence is still lacking.

**Table 1:** **Murine inflammatory pathways and their role in HSCs**.

Inflammatory pathways	Type of treatment	Effect on HSCs	HSC phenotype	*In vivo* transplantation?	Reference
IFN-α/β	LCMV infection	Pancytopenia	CFU-S	Yes	(32)
IFN-α/β	LCMV infection in INFα/β-R KO mice	None	CFU-S	Yes	
IFN-α	Single dose IFN-α	Proliferation	SP-LSK	No	(33)
IFN-α	Single dose IFN-α	Proliferation	LSK CD150^+^ CD34^−^	No	(34)
	3 doses (acute) *via* poly(I:C)	None	–	Yes	
	8 doses (chronic) *via* poly(I:C)	Loss of HSC activity	–	Yes	
IFN-α	1–2 dose(s) (acute) *via* poly(I:C)	Proliferation	LSK Flk2^−^ CD48^−^ CD150^+^	–	(36)
	3–15 doses (chronic) *via* poly(I:C)	Re-entry in quiescence or induced cell death upon transplantation		Yes	
IFN-γ	2 doses (acute)	Proliferation	LSK	No	(37)
IFN-γ	Coculture of MNC onto IFN-γ overexpressing stromal cells	Loss of HSC activity	–	Yes	(38)
IFN-γ	Single dose IFN-γ	Proliferation	SP-LSK		(39)
	Competitive transplantation of *Ifn*γ KO HSCs with WT HSCs	Better engraftment of IFN-γ KO HSCs	–	Yes	
IFN-γ	*Via E. muris* infection	Proliferation	LSK CD150^+^ CD34^−^ CD135^−^	–	(40)
		Loss of HSC activity	–	Yes	
IFN-γ	IFN-γ *in vitro*, 7 days	Reduced HSC maintenance	Lin-c-kit^+^ CD48^−^ CD150^+^	Yes	(41)
	*In vitro*, 4 days, and *in vivo via* LMCV infection	Impairs HSC proliferation		Yes	
IFN-γ	*Lgr47* KO mice	Reduction of HSC numbers	SP or LSK	Yes	(43)
	5-FU or mycobacterium infection of *Lgr47* KO mice		SP Sca-1^+^	Yes	
TNF-α	Coculture with CD8^+^ cells	Enhance LTC-IC and engraftment of HSCs	LSK	Yes	(53)
TNF-α	Competitive transplantation of WT HSCs and *Tnfrs1a* KO, *Tnfrs1b* KO, and double KO HSCs	Better engraftment of *Tnfrs1a/b* double KO HSCs	LSK Flt3^−^	Yes	(54)
	3 doses TNF-α	Loss of HSC activity	–	Yes	
TNF-α	*Tnfrs1a* KO mice	Decreased HSC number, loss of HSC activity	LSK CD34^−^	Yes	(55)
IL-1	8 days IL-1β in liquid culture	Increase proliferation and myeloid output	LSK Flk2^−^ CD48^−^ CD150^+^	–	(79)
	Chronic (20 days) IL-1β exposure	Impaired HSC activity	–	Yes	
IL-3	*Ex vivo* culture	Expansion of HSCs	LSK or LSK CD34^−^	Yes	(80)
IL-27	*Ex vivo* culture (with SCF)	Expansion of HSCs, myeloid differentiation	LSK or LSK CD34^−^ CD150^+^	No	(81)
G-CSF	*Cfsr3* KO mice	Reduction of HSPCs upon transplantation into WT animals	LSK	Yes	(98)
G-CSF	G-CSF treatment *in vivo*	Activation of dormant HSCs	–	No	(95)
G-CSF	G-CSF treatment *in vivo*	Increased HSC proportion in endosteal niches	LSK CD48^−^ CD150^+^	No	(91)
G-CSF	G-CSF treatment *in vivo*	Increased HSC proportion, impaired repopulation capacity	LSK CD48^−^ CD41^−^ CD150^+^	Yes	(92)
TLR2/TLR4	LPS/Pam3CSK4, treatment *in vitro*	Skewed myeloid differentiation	LSK Flk2^−^ IL7Ra^−^	No	(63)
TLR4	LPS treatment (4–6 weeks)	Increased HSC proportion, loss of self-renewal, myeloid differentiation	LSK Flk2^−^ CD48^−^ CD150^+^	Yes	(64)
TLR	Infection *Pseudomonas aeruginosa*	Expansion of HSCs, impaired repopulating capacity	LSK	Yes	(66)
TLR	LPS 4 doses	Increased HSC repopulating capacity	–	Yes	(67)
TLR2/TLR4/MyD88	Pam3CSK4, LPS, or ODN treatment of chimeric mice	Differentiation of donor WT cells into macrophages	–	Yes	(68)
TLR2/TLR4	LPS/Pam3CSK4, treatment *in vitro*	ST-HSCs and MPP cells produce cytokines through NF-κB	ST-HSCs (LSK CD34^+^ Flk2^−^), LT-HSCs (LSK CD34^−^ Flk2^−^)	No	(65)

*5-FU, 5-fluoruracil; CFU-S, colony-forming unit in the spleen; KO, knockout; LCMV, lymphocytic choriomeningitis virus; LPS, lipopolysaccharide; LSK, Lineage^+^ Sca-1^+^c-kit^+^; MNC, mononucleated cells; MPPs, multipotent progenitors; ODN, synthetic oligodeoxynucleotides; Pam3CSK4, synthetic bacterial lipoprotein; Poly(I:C), polyinosinic:polycytidylic acid (synthetic dsRNA); SP, side population; ST-HSCs, short-term HSCs; WT, wild type*.

## Tumor Necrosis Factors

Tumor necrosis factor alpha (TNFα) is a pro-inflammatory cytokine mainly produced by activated macrophages, lymphocytes, and endothelial cells (ECs). TNFα is synthesized as a prohormone and can be secreted after a cleavage process as a mature protein (45). Albeit it was first identified as a protein capable of inducing tumor cell necrosis (46), today it is also known that it functions as part of a number of different processes, ranging from cell apoptosis (47) to fever induction (48). TNFα signals through two different receptors, one of them being constitutively and almost ubiquitously expressed (p55 receptor, also known as TNFR1) and the other restricting its expression to hematopoietic cells (p75 receptor, also known as TNFR2) (49).

## The Effect of TNFα on Embryonic Hematopoiesis

Recently, Espín-Palazón et al. clarified the role of TNFα in zebrafish embryonic hematopoiesis (50). Based on previous observations that this specific cytokine is necessary for embryonic blood vessel development (51) and the arterial origin of HSCs, Espín-Palazón et al. attempted to investigate a possible direct correlation between HSC emergence and TNFα signaling. By means of morpholino-mediated knockdown, at a dose that led to no vascular abnormalities, they showed that the number of *cmyb*^+^ cells in the dorsal aorta at 48 hpf was significantly reduced in *tnfa*- and *tnfr2-*, but not in *tnfr1*-deficient embryos (50). This indicates that TNFα signaling is required for HSC specification, and this effect is mediated through the hematopoietic-restricted Tnfr2. The source of TNFα was shown to be primitive neutrophils, answering the question about the role of these cells during early development, when their environment is relatively sterile.

Interestingly, ectopic provision of Notch signaling resulted in rescue of HSCs in both a *tnfa*- and a *tnfr2*-deficient background, thereby proving that Notch signals downstream of TNFα. Since the main mediator of TNFα is NF-κB, the authors asked whether NF-κB is required for HSC emergence. Blocking NF-κB resulted in loss of HSCs at 48 hpf and its function was suggested to lie downstream of Tnfr2, thus linking, for the first time, NF-κB with HSC emergence.

The absence of T lymphocytes by 4–5 dpf (days post fertilization) in a *tnfa*/*tnfr2*-deficient background, stresses the importance of TNF signaling not only in HSC emergence but also in maintenance of nascent HSC fate, exactly like IFN-γ does not only affect the EHT process but also has a long-term effect on HSC repopulating activity. Moreover, cooperative functions among pro-inflammatory cytokines during embryonic hematopoiesis might exist, since knockdown of both *tnfa* and *ifng* leads to a greater reduction of *runx1* expression than the individual knockdown of either gene at 33 hpf (18). Interestingly, neither of the two cytokines was reported to have an influence on the primitive hematopoietic wave, implying that the role of primitive hematopoietic cells lies, at least partially, on the production of a variety of signaling molecules that will induce the subsequent definitive wave.

## The Effect of TNFα on Adult Hematopoiesis

Pioneer studies in human cells have shown that TNFα treatment could inhibit the proliferation of hematopoietic progenitors *in vitro* (22, 25, 30). Dybedal et al. have shown that human HSPCs (CD34^+^ CD38^−^) exposed to TNFα could not repopulate NOD-SCID mice upon transplantation (52). Moreover, they have shown that TNFα promoted myeloid differentiation rather than apoptosis of HSPCs. Several *in vivo* studies in mice have clearly identified TNFα as an important physiologic regulator of hematopoiesis. However, some discrepancies still exist in the literature regarding the role of this cytokine in HSC activity. For instance, *in vitro* assays have shown that TNFα production by CD8^+^ cells enhanced the function of HSPCs, notably by suppressing apoptosis (53). Additionally, the authors reported that, upon transplantation, hematopoietic cells exposed to CD8^+^ produced-TNFα demonstrated a better engraftment (53). However, a recent study identified TNFα as a suppressor of HSC activity *in vivo*. The authors carefully analyzed the repopulating capacity of HSCs isolated from *Tnfrs1a*^−/−^, *Tnfrs1b*^−/−^, and double knockout mice. They observed that, although the proportion of HSCs (LSK Flk2^−^) was comparable to wild type animals, *Tnfrs1a*^−/−^ or *Tnfrs1b*^−/−^ HSCs had an increased repopulation capacity and increased self-renewal activity, as demonstrated by serial transplantation experiments (54). This phenotype was even more pronounced in double knockout animals. Furthermore, they demonstrated that treatment of wild type mice with TNFα (three doses) was sufficient to suppress HSC proliferation and repopulating capacity. Nevertheless, it seems that the effect of TNFα may differ with age. Indeed, Rebel et al. noticed that the percentage of BM HSCs (LSK CD34^−^) was fourfold decreased in older (6 months) *Tnfrs1a*^−/−^ mice. The impaired self-renewal activity of *Tnfrs1a*^−/−^ HSCs was confirmed after secondary transplantation assays (55).

As stressed in this section, the effects of TNFα on HSCs are somehow complex. The comparison of the proportion of HSCs from one study to another is hampered by the use of different markers to characterize the HSC pool (see Table 1). However, it is clear that TNFα might act as a suppressor of HSC activity *in vivo*, and that this role might change in older mice. Therefore, it seems that a baseline TNFα signaling is crucial for the maintenance of HSC function through aging, providing perhaps adequate stimulation for proper HSC cell division, and hence, maintenance.

In addition to TNFα, other TNF-associated molecules have been implicated to HSC survival. Depletion of the receptor-interacting serine/threonine-protein kinase 1 (RIPK1) stimulates pro-inflammatory cytokines and results in hematopoietic cell death that is partially rescued by simultaneous deletion of *Ripk3*. Fetal liver cells from these mice fail to reconstitute lethally irradiated recipients (56). The NF-κB-associated deubiquitinase CYLD together with its substrate TRAF2 regulate HSC cycling and promote dormancy (57).

Although the role of TNF, especially during HSC maintenance and stress, is still somewhat contradictory, these studies represent a perfect example of how a classic inflammatory signaling can play multiple roles during development and adulthood. The next step is to tightly titrate both the dosage and the timing of TNF to better understand its implication in HSC biology.

## Toll-Like Receptors

Toll-like receptors (TLRs) are a family of transmembrane pattern recognition receptors that recognize a wide variety of pathogen- and danger-associated molecular patterns (PAMPs/DAMPs). TLRs are located either on the plasma membrane or in endosomes, and signal through either MyD88-dependent or TRIF-dependent pathways to activate NF-κB, IRF7, or IRF3 and induce the expression of pro-inflammatory cytokines (58, 59).

## Toll-Like Receptor Influence on Embryonic Hematopoiesis

Regarding embryonic hematopoiesis in zebrafish, it was shown by He et al. that both *tlr4bb* and *myd88* morphants demonstrated a reduced expression of the HSPC marker *runx1* at both 24 and 36 hpf and of the T cell marker *rag1* at 4 dpf, compared to their wild type siblings. To confirm their observations, they used the clustered regularly interspaced short palindromic repeats (CRISPR)/Cas9 technology and targeted gene-specific promoters involved in TLR4–MyD88 signaling. This caused a transcriptional repression of several molecules involved in the pathway and led to a remarkably decreased expression of *runx1*, while the number of *cmyb*^+^*kdrl*^+^ cells in the CHT was markedly lower compared to wild type embryos, all together depicting the importance of TLR4 signaling in HSPC emergence, in a MyD88-dependent manner (60).

Notably, morpholino-mediated knockdown of *ikbaa*, the cytoplasmic inhibitor of NF-κB, was able to rescue HSPCs in *tlr4bb* and *myd88-*deficient embryos, revealing that the impact of TLR4–MyD88 axis on HSPC emergence is mediated *via* NF-κB activation. Moreover, Notch signaling, which had already been reported to integrate with TLR signaling (61) and act upstream of Runx1 to contribute to HSPC specification (62), was proven to be impaired in the AGM region of TLR4–MyD88-deficient embryos, as reflected by the decreased expression of its target genes. Endothelial-specific Notch overexpression managed to rescue HSPCs in these embryos, showing that TLR4–MyD88–NF-κB-mediated inflammatory signaling is upstream of endothelial Notch signaling and that they, in tandem, regulate HSPC emergence during zebrafish embryonic development.

In the same study, the effect of TLR4–MyD88–NF-κB-mediated inflammatory signaling was shown to be conserved in mouse. Immunofluorescence and qRT-PCR analysis of the AGM region of *tlr4*^−/−^ mice, demonstrated a decreased expression of *Runx1* at E10.5, *cmyb, Il1b*, and *Il6* (NF-κB targets), as well as Notch target genes. Importantly, transplantation assays aiming to evaluate HSC activity, showed that 5/6 recipients transplanted with cells deriving from *Tlr4*^+/−^ embryos were reconstituted after 2 months, while only 1/6 recipients transplanted with cells deriving from *Tlr4*^−/−^ embryos was reconstituted (60).

## Toll-Like Receptor Influence on Adult Hematopoiesis

Recent studies reveal that TLR ligands can exert a direct effect on HSC activation and hematopoietic potential. Nagai et al. have found that murine HSCs (LSK Flk2^−^ IL7Ra^−^) express TLR-2 and TLR-4 and functionally respond to receptor stimulation *in vitro* (LPS and Pan3CSK4, respectively) resulting in increased myeloid differentiation and enhanced cell cycling *via* MyD88 (63). The same group addressed the consequences of *in vivo* exposure to LPS. Mice injected with LPS for 4–6 weeks harbored a higher proportion of HSCs (LSK Flk2^−^ CD48^−^ CD150^+^) compared to control mice. Moreover, upon competitive transplantation assays, HSCs activity of LPS-treated mice showed a skewing toward myeloid differentiation and impaired self-renewal capacity as compared to untreated mice (64). Interestingly, the authors also observed that LPS-treated HSCs developed some features of aged HSCs, i.e., CD150^+^ expansion and lack of both CD86 (B7.2) and CD18 (Integrin β-2) (64). In a recent study, Zhao et al. have shown that HSPCs respond to TLR stimulation by producing (*via* NF-κB activation) cytokines to regulate myeloid differentiation (65). Another study of severe sepsis reported that, although *Pseudomonas aeruginosa* caused the expansion of HSPCs (LSK) and impaired repopulating capacity, they observed a block of myeloid differentiation (66). Another group, however, suggested a different impact of TLR signaling on HSCs. They described that short-term treatment with higher-dose LPS (four doses of 35 μg, every 2 days) led to increased HSC repopulating capacity with no lineage bias (67). Thus, the dose and duration of TLR stimulation may have different outcomes on HSC activity, mainly promoting HSPC cycling and enhancing myeloid differentiation. However, despite the fact that HSPCs express TLRs, it was not clear yet whether direct recognition of pathogens by HSPCs, or changes in the microenvironment during infection were responsible for the changes in hematopoiesis. To tackle this question, Megías et al. have used an elegant experimental approach. They isolated wild type HSCs (LSK IL7Ra^−^) and transplanted them into lethally irradiated *Tlr2*^−/−^, *Tlr4*^−/−^, or *MyD88*^−/−^ recipient mice, which where then challenged with TLR agonists (Pam3CSK4, LPS, or ODN, respectively). As recipient mice could not recognize the TLR agonist injected, secondary effects of cytokines or soluble factors secreted by the microenvironment were excluded. The authors observed that wild type donor cells rapidly differentiated into macrophages, supporting the initial idea that direct stimulation of HSPCs *via* TLRs occurs *in vivo* (68). Other TLRs might very well be expressed also by HSCs. It was notably shown that TLR-9 transcripts (predominantly expressed by lymphoid progenitors) were detected in HSCs (Lin^−^Rag1^GFP−^c-kit^hi^Sca1^+^Thy1.1^lo^) (69). Last, it will be interesting to use such TLR knockout mice, in order to determine whether TLR signaling also contributes to the maintenance of HSCs in absence of infection.

## Interleukins

Interleukins (ILs) are secreted proteins produced by various immune cell types, ranging from macrophages to T lymphocytes, but also other cell types, like ECs, and their role is to orchestrate the immune system responses (70). Among all members, IL-1 and IL-3 have been shown to also play a role in hematopoiesis. The correlation between IL-1 and hematopoiesis emerged by the expression of this cytokine and its receptor, IL-1RI, in HSPCs of the BM (71–73). After early studies published from the lab of Hal Broxmeyer, it became evident that IL-1 would play synergistic roles with other factors to promote the proliferation and survival of more committed progenitors, in both mouse and humans (74, 75).

## Effects of Interleukins During Embryonic Hematopoiesis

In zebrafish, it has been reported that overexpression of *il1b* can rescue the HSPC emergence defect in TLR4-deficient embryos, while downregulation of *il1b* expression in an NF-κB-deficient background can be rescued by knocking down *ikbaa*. Therefore, IL-1β signaling appears to be downstream of the TLR4–MyD88–NF-κB-mediated regulation of HSPC emergence (60).

When Elaine Dzierzak and her group tackled the role of IL-1 in mouse embryonic development of HSCs, they discovered that IL-1R1 was detected in cells of the ventral part of the dorsal aortas from E11 onward. *Il1r1*^−/−^ embryos demonstrated higher percentage of myeloid progenitor cells (CFU-G and CFU-M), indicating that IL-1RI-mediated signaling in the AGM region limits the growth of these particular progenitors. Upon transplantation, they observed a slight decrease in repopulating cells isolated from *Il1r1*^−/−^ embryos. However, the fact that the majority of IL-1-signaling molecules were not detected in the AGM at E10 indicates that IL-1 probably does participate in HSC emergence (76).

Since IL-3 was known to be a Runx1 target, the same group also attempted to decipher the role of IL-3 in mouse embryonic hematopoiesis (77). The expression of the cytokine and its receptor was detected in major embryonic vessels at E11. Functional experiments divulged that, even if *Runx1*^+/−^ embryos have fewer HSCs in the AGM region (78), IL-3 treatment managed to rescue (and even amplify) the number of HSCs as shown by transplantation experiments, suggesting that IL-3 signaling acts downstream of Runx1 (77).

## Effects of Interleukins on Adult Hematopoiesis

Recently, the role of chronic exposure of HSCs to IL-1 was delineated in a study by Pietras et al. (79). This study showed that IL-1 exposure of HSCs leads to the activation of a Pu1-dependent program, which in turn results in HSC cycling and massive differentiation toward myeloid cells. In addition, HSCs lose their repopulation capacity. However, these results are transient, since withdrawal of IL-1 restores a normal phenotype (79). Although conflicting evidence exists, IL-3 was also shown to be capable of supporting HSC expansion and long-term multilineage repopulating capacity (80). Finally, a recent report showed that IL-27 exposure leads to expansion of HSPCs (LSK) while simultaneously promoting their differentiation toward myeloid cells (81).

It is evident that ILs play new exciting roles in the biology of HSCs, however, more studies are needed to clearly dissect the role of this family. It would be interesting to see how different ILs or combinations of different family members interact and affect HSC development and maintenance.

## Granulocyte Colony-Stimulating Factor

Granulocyte colony-stimulating factor (G-CSF) is a glycoprotein that signals through its cognate receptor, granulocyte colony-stimulating factor receptor (G-CSFR). G-CSF plays important roles in hematopoiesis, notably by inducing the production of granulocytes in response to infection (82). G-CSFR is mainly expressed in hematopoietic cells, and its stimulation leads to activation of downstream intracellular signaling cascades, including the JAK/STAT/SOCS pathway [reviewed in Ref. (83)]. Besides its role in granulopoiesis, G-CSF is used for clinical purposes to mediate HSPC mobilization from the BM into the circulation. Indeed, early reports have shown that G-CSF significantly increased the number of HSPCs in the circulation (84). This potential was confirmed by others (85), and G-CSF has been adopted as a source of HSC for transplantations, ever since.

## Effects of G-CSF During Embryonic Hematopoiesis

Initial studies to investigate the role of G-CSF during embryonic development were conducted in zebrafish. The authors showed that in a context of emergency granulopoietic response (LPS stimulation), Gcsf signaling was critical to increase the number of neutrophils in both AGM and CHT regions. However, knockdown of *gcsfr* did not impair definitive hematopoiesis (86). In a later study though, Hall and colleagues observed that emergency granulopoiesis (induced by bacterial infection) led to an increase in HSC numbers (87). This increase in HSCs was more likely due to increased cell division rather than increased EHT, and induced in a C/ebpb-driven Nos2a-dependent manner (87). This proliferative signal was confirmed by gain-of-function experiments. Both G-CSFR ligands (Gcsfa and Gscfb) caused the expansion of *runx1*^+^ HSPCs at 24 hpf. Conversely, morpholino-mediated knockdown of *gcsfr, gcsfa*, or *gcsfb* significantly reduced the number of *runx1*^+^ HSPCs (88). Moreover, it seems that HSPCs in the CHT were also greatly increased by enhanced Gcsf signaling at 48 hpf, indicating that both ligands play a role in the expansion of HSCs in the zebrafish embryo (88). Unlike in the zebrafish model, there is a lack of evidence in the literature for the involvement of G-CSF or G-CSFR (*Csfr3*) in promoting HSC expansion during embryonic development in mice.

## Effects of G-CSF on Adult Hematopoiesis

Both *Gcsf*^−/−^ and *Csfr3*^−/−^ animals are viable, despite having reduced number of circulating neutrophils (89, 90). G-CSF administration *in vivo* was reported to increase the proportion of HSCs (LSK CD150^+^ CD48^−^) in the central marrow, but not in endosteal niches in the BM (91). More recently, Schuettpelz and colleagues confirmed the increase in the pool of HSCs (LSK CD150^+^ CD48^−^ CD41^−^) in the BM upon G-CSF treatment, together with loss of long-term repopulating activity due to induction of TLR signaling in HSCs. Interestingly, loss of TLR signaling or suppression of commensal flora (*via* antibiotic treatment or housing in a germ-free environment), mitigates the G-CSF-mediated expansion of HSCs (92).

Granulocyte colony-stimulating factor is also largely used as a mobilizing agent, notably by targeting cells composing the HSC niche, and critical for HSC maintenance (93). It is important to note that HSC mobilization by G-CSF is abolished in *Csfr3*^−/−^ mice (94) and G-CSF administration is capable of activating dormant HSCs *in vivo* (95). However, chimeric mice harboring mixtures of *Csf3r*^+/+^ and *Csfr3*^−/−^ hematopoietic cells have normal mobilization. This emphasizes that G-CSFR expression in HSCs is not required for G-CSF-mediated mobilization, and probably acts in trans on HSCs. This study concluded that G-CSFR expression on HSCs or stromal cells is not important for mobilization. Rather another unknown population of G-CSFR expressing cells is responsible for acting on HSC mobilization (94). Interestingly the Manz lab showed that at least in situations of systemic bacterial stress, Tlr4-expressing ECs are the source of G-CSF and contribute to emergency myelopoiesis (96, 97). The baseline numbers of HPSCs in *Csfr3*^−/−^ mice was estimated to be similar to *Csfr3*^+/+^ controls, although the authors used long-term culture-initiating cells (LTC-ICs) to draw this conclusion. However, by competitive transplantation assays, the authors noticed a marked reduction of *Csfr3*^−/−^ HSPCs (LSK) frequencies in recipient mice (98).

## Macrophage Colony-Stimulating Factor

Macrophage colony-stimulating factor (M-CSF) is a growth factor that is crucial for the proliferation, differentiation, and survival of monocytes and macrophages but also progenitor cells in the BM. Combination of human or mouse G-CSF, GM-CSG together, or with addition of IL3 led to increased cycling and elevated numbers of hematopoietic progenitors *in vitro* (99–101). The lab of Michael Sieweke brought evidence that M-CSF could exert a function directly on HSCs. Based on the observation that the monocyte/macrophage transcription factor MafB was highly enriched in HSCs (LSK CD34^−^Flk2^−^), but not in downstream progenitors, Sarrazin and colleagues investigated the effect of MafB deficiency on HSCs. *MafB*^−/−^ HSCs were more sensitive to M-CSF treatment, and as a result, more prone to skewing toward myeloid differentiation (102). In a recent study, it was shown that M-CSF could directly induce endogenous PU.1 protein (a transcription factor promoting myelomonocytic differentiation), in single HSCs *in vivo* and stimulates a reversible, PU.1-dependent myeloid differentiation (103).

## Inflammation and Hematological Diseases

Based on the numerous connections between inflammation and hematopoiesis, it should be unsurprising that deregulation of this interplay plays a role in hematopoietic diseases. Below, we will briefly discuss some examples of HSC-associated diseases, and how inflammation affects the emergence, progression, or the outcome of the disease.

## Myelodysplastic Syndromes

Myelodysplastic syndromes (MDS) (104) are a heterogeneous group of blood diseases mainly characterized by ineffective hematopoiesis, generic blood dysplasia, and a tendency toward leukemic transformation. The pathophysiology lying behind the progression of such diseases is extremely variable and, therefore, classified in a generic way, involving causes related to intrinsic abnormalities in myeloid progenitors and causes related to extrinsic alterations in the BM microenvironment (104, 105). Although the precise connection is still being elucidated, several studies have correlated MDS with inflammatory disorders. In a recent study, 4.4% of MDS patients presented also autoimmune manifestations (106). In addition, it was reported that inflammatory bowel disease patients were diagnosed with MDS. Both these studies suggest an association between MDS and inflammation (107). However, it is still not clear whether inflammation precedes MDS or vice versa.

Toll-like receptor signaling is one of the pathways identified as a connection between inflammation and MDS. In particular, a number of TLRs and several signal transducers involved in this pathway have been found overexpressed in a high percentage of MDS patients (108). For example TLR2 and TLR9 are overexpressed in all MDS subtypes. Chronic TLR stimulation in mice induces phenotypes very similar to MDS (64). Among all TLRs that were found upregulated (109), Wei et al. reported a somatic mutation of TLR2, namely TLR2-F217S, present in 11% of MDS patients and connected with amplified NF-κB activation, a transcription factor that had already been associated with the disease (108).

NF-κB was one of the factors expected to participate in those diseases due to its important role in HSC proliferation and differentiation (110). Inhibition of its transcriptional activity was reported to result in apoptosis in both healthy and MDS BM progenitors, indicating its high significance in the maintenance and progression of the disease (111–113). Although induction of NF-κB alone is not sufficient to cause MDS, deregulation of NF-κB in the non-hematopoietic compartment can cause a myeloproliferative disorder (114).

Apart from NF-κB, TLRs are also known to induce IFN production. In MDS patients, IFN secretion levels were found severely elevated, which seems to strongly impair hematopoietic progenitor differentiation, for instance, erythroid differentiation (115) and reduce their long-term repopulating activity (116). Many other cytokines are induced by TLR signaling and also play a role in MDS. For example, TLR4 contributes to elevated TNFα levels in MDS patients (117).

The BM microenvironment plays a crucial role in MDS and is also the source or the substrate of many cytokines produced. MDS have been strongly associated with a specific cell type known as the myeloid-derived suppressor cells (MDSCs), which are mostly known to accumulate in cancer patients and suppress effector T cell response, thereby constituting one of the greatest defenses of a tumor against the host’s immune system (118, 119). These cells were also found notably increased and accumulated in the BM of MDS patients, where they have been proposed to mediate their effect mainly *via* secretion of several cytokines (e.g., IL-10) and other inflammatory effectors [e.g., reactive oxygen species (ROS)] leading to DNA instability and constantly active inflammation, which creates a permissive environment for the development of myelodysplasia (120).

## Inherited Bone Marrow Failure

Bone marrow failure (BMF) syndromes include a group of blood disorders that can be either acquired or inherited and they are characterized by diverse kinds of defects, involving all hematopoietic lineages and lead to cytopenia. Inherited BMF syndromes have been associated with increased risks of developing MDS, acute myeloid leukemia (AML), or even solid tumors (121). This group of rare genetic disorders involves Fanconi anemia (FA), Diamond–Blackfan anemia (DBA), dyskeratosis congenita (DC), and Shwachman–Diamond syndrome (SDS). Similar to MDS, BMF syndromes have also been correlated with inflammatory signals, and this correlation is better studied in FA (122).

During the course of this disease, a cluster of 16 genes (FANC genes) coding for DNA repair proteins are genetically defective (123–125). The prominent role of FA proteins is to respond to DNA damage and DNA replication stress and lead to DNA repair (123–125). Thus, FA is a genomic instability syndrome that can lead to BMF or leukemia. Patients have been often reported to demonstrate elevated plasma or intracellular levels of TNFα (126, 127) that affects the production and secretion of other cytokines. Imbalance between different cytokines like IL-6 and GM-CSF affects the BM microenvironment (128). These alterations can cause deregulation of cellular homeostasis and are crucial for the progression of the disease (129–132).

Apart from mediating its role through regulation of other cytokine secretion, however, TNFα is also known to induce ROS, which represent another important factor for the disease. Several FA patients cannot detoxify superoxide anions, which leads to elevated oxidative stress. Indeed, Zhang et al. showed that, in a *Fancc*-deficient background, TNFα-injected mice had high levels of oxidative stress, indicating the significant role of this FA protein in oxidative stress inhibition and suggesting an interplay between FA proteins and TNFα in the disease progression (133).

Several studies have proven that one of the reasons why patients experience the TNFα upregulation is TLR signaling. In particular, *FancA*- and *FancC-*deficient mononuclear phagocytes, as well as mononuclear phagocytes deriving from FA patients, showed upregulated levels of TLR8 and its canonical downstream effectors, which is known to lead to increased TNF production (134). On the other hand, TLR8 signaling also activates IFN (116), a cytokine that has also been found upregulated in FA patients (131), linking inflammatory signaling with this disease. Finally Walter et al. showed that repeated activation of dormant HSCs due to infection or constant blood loss, leads to DNA damage. Thus HSCs lacking an intact FA DNA repair pathway cannot overcome constant stress and, as a result, the hematopoietic system collapses completely (135).

## Leukemia

Leukemias are a group of blood cancers defined by an increased number of abnormal white blood cells. Leukemias can be either chronic or acute and can be roughly divided in four different categories: AML, chronic myeloid leukemia (CML), acute lymphoblastic leukemia (ALL), and chronic lymphoblastic leukemia (CLL), based on whether the origin of the transformed cell is myeloid or lymphoid, but also on the characteristics of the disease initiation and progression. Although these leukemias mainly refer to differentiated hematopoietic populations, AML and CML arise from HSCs. CLL can be derived from more differentiated cells but accumulating evidence shows that they may also originate, at least partly, from HSCs (136).

The significance of inflammation in leukemia progression and maintenance is not unexpected, given its known role in hematopoiesis. Apart from the correlation between inflammation and MDS or BMF, which might result in leukemic transformation, it is known today that inflammatory pathways are directly connected with blood cancers (137–139).

One of the leukemic diseases for which the connection and dependence on inflammation has been well studied is AML. AML is the most common type of acute leukemia in adults. AML phenotypes can be divided in several subtypes and is generally characterized by the presence of hyperproliferative myeloid cells (140). TNFα and IFNγ have been shown to suppress clonogenic growth and promote differentiation in blast cells from AML patients (141). Studies on AML patients showed that the canonical NF-κB pathway is constitutively active in AML cells (137). Kurokawa and his group recently showed that the NF-κB pathway was active in leukemia-initiating cells (LICs) isolated from several murine myeloid leukemia models, as well as AML patients (142). Treatment with IκB-SR, a super repressor of NF-κB, led to a significant growth delay of murine myeloid leukemic cells. Additionally, the leukemia progression after transplantation of these cells into irradiated mice was delayed, suggesting that NF-κB is a contributing factor to the disease progression, but not to the survival and fate maintenance of the leukemic cells. Analysis of published microarray-based gene expression profiles and comparison between human or murine LICs and normal HSPCs (143–145) hinted at TNFα as the factor that maintains the constitutively active NF-κB pathway. Indeed, when LICs were treated with a TNFα-neutralizing antibody, p65 nuclear translocation was markedly impaired, indicating that constitutive NF-κB activation is dependent on TNFα signaling. Furthermore, *Tnf*-deficient cells were significantly impaired in their potential to initiate leukemia, but NF-κB inhibition alone was not sufficient to inhibit leukemic progression. Thus, TNF may exert its function through other factors besides NF-κB (142). Additionally CML, ALL, and CLL cells also exhibit elevated NF-κB levels indicating a potential role of inflammatory signaling in this disease (146–150).

Other inflammatory cytokines are also prominent in leukemia (151). CLL patients exhibit elevated levels of IFN-γ, IL6, IL8, and IL10 (152–154) that can be produced by either CLL cells or from cells of the microenvironment. Many mutations on inflammatory mediators also exist in CLL patients. For example, MYD88, a mediator of toll receptor signaling, is mutated in 3–10% of patients (155, 156).

It is evident that inflammation and leukemia are heavily interconnected. However we still do not know clearly the order of events and how inflammation affects disease progression.

## Discussion and Future Directions

**Figure 1:**
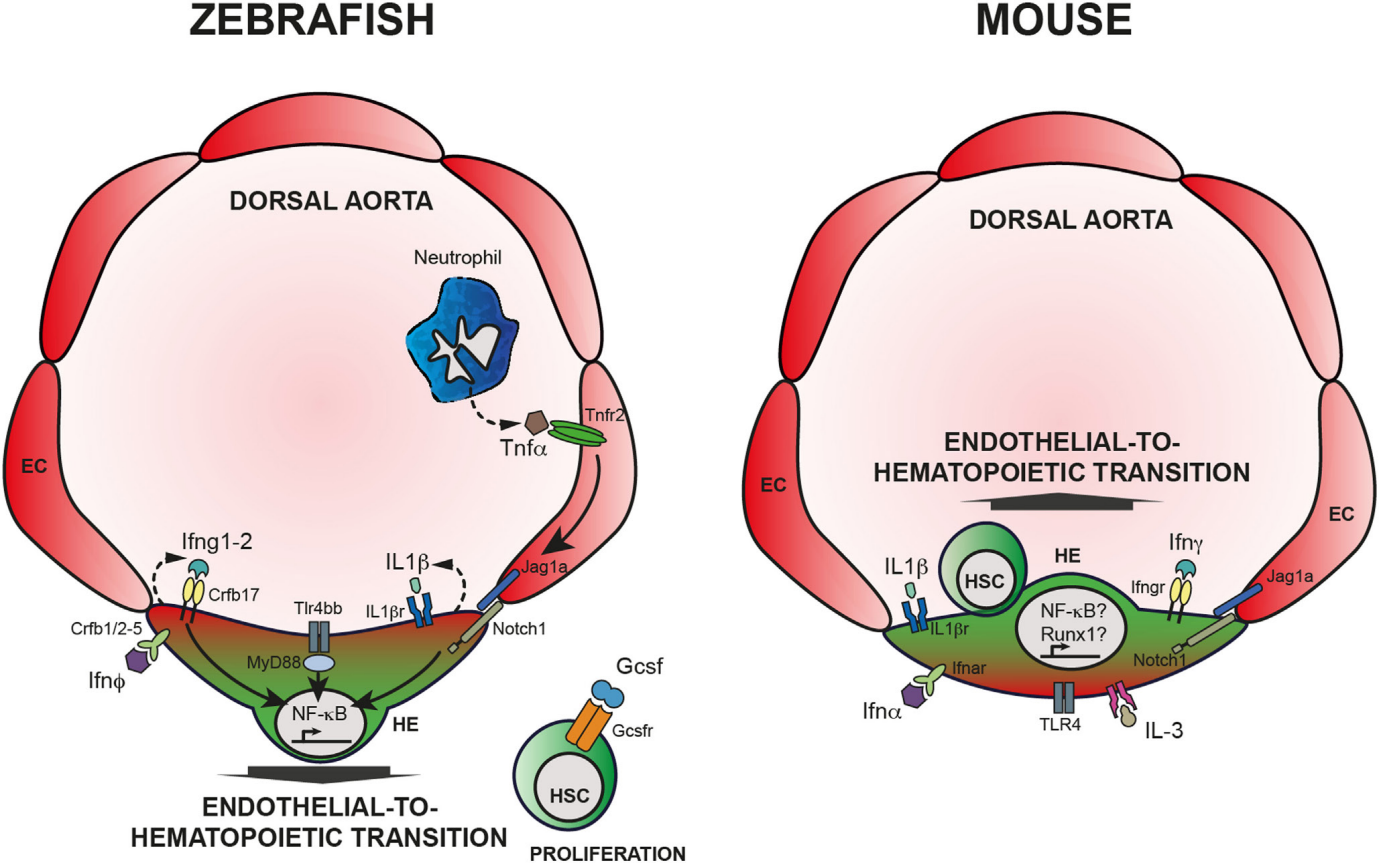
**Inflammatory signaling is required for HSC emergence**. Left panel: HSC specification in zebrafish embryos. Neutrophils produce TNFα that binds Tnfr2, expressed by endothelial cells (EC). Activation of Tnfr2 leads to upregulation of *jag1a* and activation of Notch signaling in the hemogenic endothelium (HE), ultimately leading to the activation of NF-κB. Alternatively, Tlr4/Myd88 also induce NF-κB, crucial for HSC specification throughout endothelial-to-hematopoietic transition. Downstream of Notch1 activation, Ifng participates in HSC specification in a cell autonomous fashion. IL-1β signaling, downstream of the TLR4–MyD88–NF-κB axis has also an impact on HSC emergence as does Ifn-ϕ. Finally, Gcsf acts on newly generated HSCs and contributes to HSC proliferation; however, the mechanism is still unclear. Right panel: HSC specification in mouse embryos. Contrary to zebrafish embryos, it is still unclear how inflammatory pathways are being regulated in the developing embryo, or what are the sources of inflammatory cytokines. Nevertheless, it is clear that IFN-α, IFN-γ, as well as Il-1β, IL-3, and TLR4 are required for proper HSC specification, possibly engaging Notch1 signaling and Runx1 and/or NF-κB.

In summary, it is by now clear that inflammatory signaling arises as a novel regulator of HSCs emergence and maintenance, both under sterile conditions and in case of stress and disease. Studies in development and during homeostasis provide evidence that HSCs are affected by alteration of different cytokines (Figure 1). Excellent reviews referring to the subject of inflammatory signaling in hematopoiesis can also complement the reading of this review (157–163). However, many questions are still unanswered and two major points include (a) the interaction of HSCs with other cells like niche and immune cells (158) and (b) the ligands that lead to inflammatory response and their source.

Diverse kinds of cells constitute the hematopoietic niche like osteoblasts, mesenchymal stem cells, ECs, and others (164, 165). For example, mesenchymal stem cells participate in the interplay with HSCs but can also interact with immune cells and adopt both pro- and anti-inflammatory roles (166–170). Immune cells have been found integral for HSCs both during development but also in adulthood. Primitive neutrophils provide crucial cytokines that are imperative for HSC formation during development (50). Macrophages play prominent roles during HSC formation in development but are also actively participating in the BM microenvironment (36, 171, 172). In depth characterization of all these interactions is needed to get a full overview of the interplay between cell populations.

The population of the niche and immune cells discussed above can actually be the source of different cytokines that will act on respective receptors on HSCs both during development and maintenance. Communication with other sources like commensal flora could also provide a source of stimulation for HSCs (92). Thus, most data to date support the hypothesis that the inflammatory ligands that affect HSCs are produced from various sources of the microenvironment. However, another hypothesis could hint to the existence of endogenous ligands that can play autoregulatory roles in HSCs. Lately, it has been shown in many systems that the epigenetic status of a cell plays crucial role in the expression of endogenous repetitive elements like endogenous retroviral elements. Members of the RIG-I-like receptors respond to this upregulation and can mediate an IFN response (173–175). It would be interesting if such a mechanism is used to fine tune the inflammatory milieu of the hematopoietic system.

Another aspect that needs further investigation is the consequence of acute versus chronic inflammatory stress on HSCs. Although many predicted that chronic stress will lead to HSC exhaustion, Pietras et al. showed that HSCs re-enter quiescence in conditions of chronic stress (36). However, upon a second challenge, these HSCs are much more vulnerable. Could an epigenetic memory, newly established on the challenged HSCs, lead them to such a reaction?

Finally, understanding the inflammatory pathways in a disease context is of crucial importance, since this knowledge has often provided us with the potential to target these pathways and treat the diseases. To name just few examples, specific inhibition of TLR2 and its downstream effectors showed significant results in MDS patients since it induced differentiation, apoptosis, and impaired the clonal potential of MDS cells. OPN-305, an antibody against TLR2, is entering phase II clinical trials and holds promise for MDS treatment (176). Monoclonal antibodies against CD33 [gemtuzumab ozogamicin (177)] and bispecific antibodies like AMG330 (anti CD33 and CD3) are being explored for AML therapy (178). Both these antibodies redirect cytolytic effector T cells against leukemic cells.

In short, inflammatory signaling has a diverse and multifaceted role in many aspects of HSC biology from the generation of HSCs to their expansion and maintenance, response to stress, and disease. Various groups have contributed to our understanding of these non-immune roles of inflammatory agents, but many questions remain still unanswered.

## Author Contributions

TC, SL, and ET wrote the manuscript; TC designed the figure and the table.

## Conflict of Interest Statement

The authors declare that the research was conducted in the absence of any commercial or financial relationships that could be construed as a potential conflict of interest.
